# Variation for Composition and Quality in a Collection of the Resilient Mediterranean ‘de penjar’ Long Shelf-Life Tomato Under High and Low N Fertilization Levels

**DOI:** 10.3389/fpls.2021.633957

**Published:** 2021-04-07

**Authors:** Elena Rosa-Martínez, Ana M. Adalid, Luis E. Alvarado, Resurrección Burguet, María D. García-Martínez, Leandro Pereira-Dias, Cristina Casanova, Elena Soler, María R. Figàs, Mariola Plazas, Jaime Prohens, Salvador Soler

**Affiliations:** ^1^Instituto de Conservación y Mejora de la Agrodiversidad Valenciana, Universitat Politècnica de València, Valencia, Spain; ^2^Meridiem Seeds S.L., Torre-Pacheco, Spain

**Keywords:** *Solanum lycopersicum*, plant breeding, local varieties, metabolites, taste, nutritional quality, abiotic stress

## Abstract

The ‘de penjar’ tomato (*Solanum lycopersicum* L.) is a group of local varieties from the Spanish Mediterranean region carrying the *alc* mutation, which provides long shelf-life. Their evolution under low-input management practices has led to the selection of resilient genotypes to adverse conditions. Here we present the first evaluation on nutritional fruit composition of a collection of 44 varieties of ‘de penjar’ tomato under two N fertilization levels, provided by doses of manure equivalent to 162 kg N ha^–1^ in the high N treatment and 49 kg N ha^–1^ in the low N treatment. Twenty-seven fruit composition and quality traits, as well as plant yield and SPAD value, were evaluated. A large variation was observed, with lycopene being the composition trait with the highest relative range of variation (over 4-fold) under both N treatments, and significant differences among varieties were detected for all traits. While yield and most quality traits were not affected by the reduction in N fertilization, fruits from the low N treatment had, on average, higher values for hue (5.9%) and lower for fructose (−11.5%), glucose (−15.8%), and total sweetness index (−12.9%). In addition, lycopene and β-carotene presented a strongly significant genotype × N input interaction. Local varieties had higher values than commercial varieties for traits related to the ratio of sweetness to acidity and for vitamin C, which reinforces the appreciation for their organoleptic and nutritional quality. Highest-yielding varieties under both conditions displayed wide variation in the composition and quality profiles, which may allow the selection of specific ideotypes with high quality under low N conditions. These results revealed the potential of ‘de penjar’ varieties as a genetic resource in breeding for low N inputs and improving the organoleptic and nutritional tomato fruit quality.

## Introduction

Use of nitrogen (N)-enriched fertilizers has sharply escalated since the Green Revolution and has allowed dramatic increases in crop yields. However, severe impacts of overfertilization on the environment forced governments to implement sustainable agriculture policies (Zhang et al., 2015). The current environmental situation has prompted research studies to understand the effects of decreasing N inputs on different crops and the development of new varieties with improved N use efficiency. In general terms, N shortage is associated with a limitation of plant growth, photosynthetic rate, and synthesis and accumulation of bioactive compounds in fruits.

Tomato (*Solanum lycopersicum* L.) is the second vegetable in acreage after onions (FAOSTAT, 2020). Official recommendation of N inputs for intensive tomato cultivation varies between 200 and 240 kg N ha^–1^ in open field and between 380 and 410 kg N ha^–1^ in greenhouse (Ramos and Pomares, 2010). However, in the last decades, growers have been supplying nitrogen fertilizers well above those requirements (Thompson et al., 2007). Studies about the N supply effects on tomato showed that yield increased with N fertilization until a certain level, above which N had no longer a positive effect on yield, and even decreased it (Elia and Conversa, 2012; Djidonou et al., 2013). On the other hand, controversial responses to low N inputs are reported regarding tomato fruit quality (De Pascale et al., 2016; Truffault et al., 2019; Hernández et al., 2020).

Within the broad array of diversity of tomato types, there is a group of varieties (known as ‘de penjar’) which could potentially constitute a genetic resource in breeding for low N inputs. The ‘de penjar’ tomato is distinctively characterized by the presence of the *alc* mutation, which is associated with long shelf-life (LSL) of fruits, up to 6–12 months after harvest (Casals et al., 2012). The ‘de penjar’ (literally meaning “for hanging”) type of tomato is mainly composed of local varieties preserved by generations of small farmers. Plants of ‘de penjar’ tomato generally produce round to flat, medium-sized fruits (30–90 g) with higher acidity and soluble solid content than standard tomato; however, the ‘de penjar’ varieties display a great variability in morphoagronomic and fruit quality characteristics, according to their traditional area of cultivation and to their uses (Cebolla-Cornejo et al., 2013; Casals et al., 2015; Figàs et al., 2015). Historically, ‘de penjar’ tomatoes have been cultivated in open field and under rain-fed, low-input conditions (Conesa et al., 2020). This has led to the selection for resilient varieties. The drought tolerance of LSL varieties is well documented and has been studied in recent years (Fullana-Pericàs et al., 2019). However, to our knowledge, scarce information is available on nitrogen fertilization for ‘de penjar’ or other LSL tomato cultivation. Some conducted trials indicate that N needs of ‘de penjar’ tomato are around 170–180 kg ha^–1^ (Seda and Muñoz, 2011), far below the N fertilization levels required for intensive standard tomato cultivation. Considering that ‘de penjar’ tomato has evolved under low input management practices and its already reported drought tolerance, we hypothesize that it could also show resilience against other abiotic stresses, such as low nitrogen supply.

In the present work, we evaluated a collection of 39 local varieties and 5 commercial varieties of ‘de penjar’ tomato, grown under two N levels, for agronomic and nutritional fruit composition traits. The aim was to provide information on the existing variability and behavior of these materials under different N supply conditions and to draw conclusions on the effect of the reduction of N inputs on their fruit quality and composition.

## Materials and Methods

### Plant Material and Cultivation Conditions

A collection of 44 varieties of ‘de penjar’ tomato from different origins throughout the Valencian Region, located on the Spanish eastern coast, were evaluated under two N fertilization conditions. The collection was composed of 39 local varieties and 5 commercial varieties. Passport data on each of the accessions used is included as Supplementary Table 1.

Plants were grown during the spring–summer season of 2019 in two neighboring open-field plots located in Alcossebre (Castelló, Valencian Region; GPS coordinates of the field plots: 40°13″21′ N 0°15″51′ E). Both field plots were certified for organic farming and had followed the same agricultural practices for the last 5 years. Similar crop management practices and fertilization were applied to both field plots, except for the N fertilization level. One field plot was submitted to a N fertilization dose of 162 kg N ha^–1^, equivalent to the levels commonly provided in the cultivation of ‘de penjar’ tomato (Seda and Muñoz, 2011). This dose has been considered in the present work as high N fertilization treatment (HN). For the other field plot, a dose of 49 kg of N ha^–1^ (i.e., 30.2% of the HN) was applied. This dose has been considered in the present work as low N fertilization treatment (LN). According to the organic farming practices that have been followed in the experiment, certified organic fertilizers were used. An organic basal dressing consisting of sheep manure (Organia, Fertinagro, Teruel, Spain) was applied in both field plots, shortly before cultivation, to provide the desired levels of N fertilization. Besides, the LN was supplemented with P (Fosfoser ECO GR, Mapryser, Barcelona, Spain) and K (Summum Líquido Quality 0-0-15, Fertinagro, Teruel, Spain), to equal the quantities of P and K present in the manure of the HN treatment (60 kg P_2_O_5_ ha^–1^ and 174 kg K_2_O ha^–1^). Both P and K fertilizers in the LN were supplied with the irrigation system. Since fertilization in the form of manure is characterized by a slow release of nutrients, the total P and K fertigation was distributed on a fortnight basis. Plantlets were transplanted at the five true-leaf stage on May 8th 2019. Plants in both field plots were irrigated throughout the entire cultivation period using an exudation irrigation system (16 mm; Poritex, Barcelona, Spain), for a total volume of 127 L plant^–1^, so that the water would not be limiting in the evaluation, as confirmed by the lack of phenotypic symptoms of water stress and by the calculation of crop evapotranspiration (ETc) for ‘de penjar’ tomato. Immediately after the transplant, a watering of 5 L plant^–1^ was applied. Subsequently, 4 L plant^–1^ was applied weekly during the next 3 weeks, distributed in 2 days per week; from weeks 4 to 12, 6 L plant^–1^ was applied weekly, distributed in 3 days per week; from weeks 13 to 16, 8 L plant^–1^ was applied weekly, distributed in 4 days per week; finally, from weeks 17 to 20, plants were irrigated with 6 L plant^–1^ on a 2 days per week basis.

A total of six replicates per accession (i.e., three replicates per accession and N fertilization treatment, with two plants per replicate) were distributed in a completely randomized block design. Standard local practices for the ‘de penjar’ tomato were used during the experiment. Plants were staked with canes that were inclined so that a triangular structure was formed enabling a double-row distribution, with 3.00 and 0.80 m between wide and narrow rows, respectively. Plants were spaced at 0.35-m intervals within rows. Crop management included no pruning and manual weeding.

Climate conditions on the entire cultivation period are included as Supplementary Figure 1. Average temperature during the cultivation period varied between 15.6 and 28.1 °C. The month with the highest mean radiation was June, with 24.8 MJ m^–2^, and this parameter declined during the following months, to an average of 16.6 MJ m^–2^ in September. Pluviometry was mostly concentrated on May 24th (12.1 mm), June 5th (14.3 mm), and at the end of the cultivation period, on August 20th (23.7 mm) and September 11th (21.3 mm), 13th (14.2 mm), and 21st (36.2 mm).

### Soil Analysis

A soil physicochemical and composition analysis was performed in both field plots before the transplant and before applying the fertilization. Five samples consisting of five randomly selected spots per each of the two field plots were considered for soil analysis (*n* = 5 × 2 = 10). For each sample, five fractions of soil surrounding the selected spot, between 10 and 30 cm deep, were extracted, homogenized, and left to dry at room temperature. A portion of 500 g of each dried homogenate was used for the analysis. Physical and chemical parameters were evaluated following the procedures described in Reeuwijk (2002): particle size analysis, pH in water and pH in potassium chloride, electrical conductivity, contents in total nitrogen, carbonates, and organic matter, carbon:nitrogen ratio, and mineral contents of available phosphorus, potassium, calcium, magnesium, iron, zinc, and copper. Soil texture for both fields was clay loam, with a composition of 41% sand, 36% clay, and 23% silt (Soil Science Division Staff, 2017). There were no significant differences between the two field plots for any of the soil physicochemical and composition parameters analyzed. According to the Spanish interpretation scales for each of the different elements evaluated (Yáñez Jiménez, 1989), the soil was slightly saline and had normal contents of total nitrogen and carbonates, high presence of organic matter, and moderately high carbon:nitrogen ratio. Data of soil characteristics is included as Supplementary Table 2.

### Vegetative and Plant Trait Evaluation

A chlorophyll meter (SPAD-502, Minolta Camera Co., Osaka, Japan) was used to take SPAD values from upper fully expanded leaves. Four readings per replicate were taken. Readings were done according to manufacturer’s instructions. Total number of fruits (fn) produced per replicate was counted during the harvest period (July 15th–September 30th). Fruits at the red stage of ripeness in both N fertilization treatments were harvested once a week during this period, so that the same number of harvests was made in the two treatments. Yield was calculated from the previous data and the average fruit weight (frw) per replicate as fn × frw. In addition, a resilience index, for each accession of the 44 evaluated and trait, was calculated as the ratio between the mean value under the low N treatment and the mean value under the high N treatment (LN/HN).

### Fruit Trait Analysis

Five to ten fruits per replicate were brought to the laboratory to be processed for chemical composition analysis. The fruits were collected from the second to fifth truss at the red stage of ripeness. Before processing, five representative fruits per replicate were weighed in a digital scale in order to calculate the average fruit weight (frw). Units in which vegetative and fruit traits are expressed are included in Table 1.

**TABLE 1:** Vegetative and fruit traits evaluated in the ‘de penjar’ tomato collection, abbreviations used in tables and figures, and units in which they are expressed in the present work.

**Trait**	**Abbreviation**	**Units**
*Vegetative and plant traits*		
SPAD value	spad	−
Yield	y	kg plant^–1^
*Fruit traits*		
Fruit mean weight	frw	g
Lightness (color coordinates)	L	−
Chroma (color coordinates)	chroma	−
Hue (color coordinates)	hue	°
Fruit nitrogen content	Nf	g kg^–1^ dm^1^
Fruit carbon content	Cf	g kg^–1^ dm
pH	pH	−
Soluble solid content	ssc	%
Titratable acidity	ta	%
Citric acid content	cit	g kg^–1^ fw^2^
Malic acid content	mal	g kg^–1^ fw
Total acid (citric + malic) content	tacid	g kg^–1^ fw
Citric:malic acid ratio	citmalr	−
Fructose content	fru	g kg^–1^ fw
Glucose content	glu	g kg^–1^ fw
Total sugar (fructose + glucose) content	tsug	g kg^–1^ fw
Fructose:glucose ratio	fruglur	−
Total sweetness index	tsi	−
Soluble solid content:titratable acidity ratio	ssctar	−
Total sugar:total acid ratio	tsugtacidr	−
Total sweetness index:titratable acidity ratio	tsitar	−
Vitamin C (ascorbic + dehydroascorbic) content	vitc	g kg^–1^ fw
Lycopene content	lyc	mg kg^–1^ fw
β-Carotene content	bcar	mg kg^–1^ fw
Total carotenoid content	tcar	mg kg^–1^ fw
Glutamic acid content	gluta	g kg^–1^ fw
Aspartic acid content	aspa	g kg^–1^ fw

*^1^dm: dry matter.*

*^2^fw: fresh weight.*

#### Sample Preparation

Fruits were longitudinally cut in half, and seeds were eliminated. One half of cut tomatoes were bulked and squeezed with a domestic juice extractor for subsequent analysis of pH, titratable acidity, contents in soluble solids, reducing sugars (glucose, fructose), organic acids (citric, malic), and amino acids (glutamic, aspartic). For vitamin C analysis, 3% metaphosphoric acid (MPA) was added to the tomato liquid extract (1:1), in order to lower the pH for preventing degradation of ascorbic acid (Chebrolu et al., 2012). The other tomato halves were homogenized in liquid nitrogen using a domestic grinder, for subsequent freeze-drying. Homogenized tomato powder was used for analysis of contents in lycopene, β-carotene, total carotenoids, total nitrogen, and total carbon.

#### Soluble Solid Content, pH, and Titratable Acidity

Soluble solid content was measured using 0.5 mL of liquid extract with a HI 96801 digital refractometer (HANNA instruments, Padua, Italy). Titratable acidity and pH were measured with a PH-Matic 23 analyzer (Crison Instruments, Barcelona, Spain), from 10% (w/v) aqueous tomato extract, using NaOH 0.1 M as titrating reagent. From values of soluble solid content and titratable acidity, the ratio between those was calculated.

#### Sugars, Organic Acids, Vitamin C, and Amino Acids

Contents in reducing sugars, organic acids, vitamin C, and amino acids were measured by high-performance liquid chromatography (HPLC) using a 1220 Infinity LC System (Agilent Technologies, CA, United States) equipped with a binary pump, an automatic injector, and a UV detector. One aliquot of liquid extract per replicate was centrifuged for 5 min at 10,000 rpm, diluted with Milli-Q^®^ water (1:1), and filtered through 0.22 μm PVDF MILLEX-GV filters (Merck Millipore, MA, United States). The same sample was used to perform the analysis of sugars and organic acids, following the method indicated in Fernández-Ruiz et al. (2004), with slight modifications. Glucose and fructose separations were performed using a Luna^®^ Omega SUGAR column (3 μm; 150 × 4.6 mm; Phenomenex, CA, United States) and a refractive index detector (350 RI detector, Varian, CA, United States) coupled to the HPLC system. The mobile phase consisted of solvent A (ACN 100%) and solvent B (water). The gradient was isocratic 75% A: 25% B, and the flow rate was 1 mL min^–1^. Citric and malic acids were separated using a Rezex ROA-Organic Acid H+ (8%) column (150 × 7.8 mm; Phenomenex) and detected by HPLC-UV at 210 nm. The mobile phase consisted of an isocratic gradient of 100% 1 mM H_2_SO_4_, and the flow rate was 0.5 mL min^–1^.

From the values of contents in fructose, glucose, citric and malic acids, total sugar content and total acid content were calculated as fructose + glucose and citric acid + malic acid, respectively. In addition, the ratios fructose:glucose, citric:malic, and total sugars:total acids were determined. Total sweetness index was also calculated as (Beckles, 2012) (0.76 × [glucose]) + (1.50 × [fructose]) and used for also determining the ratio between total sweetness index and titratable acidity.

For extraction of vitamin C, one aliquot of liquid tomato extract in 3% MPA per replicate was cold centrifuged and filtered through 0.22 μm PVDF filters. In order to quantify vitamin C as the sum of ascorbic (AA) and dehydroascorbic (DHA) acids, the DHA present in each sample was reduced to AA adding 5 mM tris(2-carboxyethyl)phosphine hydrochloride (TCEP) in 1:1 proportion. The AA peak was subsequently detected and quantified by HPLC-UV at 254 nm using a Brisa “LC^2^” C18 column (3 μm; 150 × 4.6 mm; Teknokroma, Barcelona, Spain), following the method described in Chebrolu et al. (2012).

Determination of glutamic and aspartic acid contents was carried out using liquid tomato homogenate. After a previous derivatization with 30 mM 2,4-dinitro-1-fluorobenzene (DNFB) reagent at moderate basic pH and 60 °C, peak detection and analysis by reversed-phase HPLC-UV at 363 nm were performed as described by Agius et al. (2018).

#### Carotenoids

Lycopene, β-carotene, and total carotenoids were extracted using 30 mg of freeze-dried powdered material per replicate, which was incubated with ethanol:hexane 4:3 (v/v) in the darkness and shaken at 200 rpm for 1 h. Subsequently, carotenoids were quantified from UV/V spectrophotometric absorbance values at 452, 485, and 510 nm of the previously separated hexane phase, using the following equations (Zscheile and Porter, 1947):

$$\text{lycopene}=\frac{{\text{Abs}}_{510}\times 537\times 2.7}{\text{weight}\times 172}\times 100$$

$$\beta \text{-carotene}=\frac{{\text{[Abs}}_{452}-\left({\text{Abs}}_{510}\times 0.9285\right)]\times 533.85\times 2.7}{\text{weight}\times 139}\times 100$$

$$\mathrm{total}\mathrm{carotenoids}=\frac{{\text{Abs}}_{485}\times 2.7}{\text{weight}\times 181}\times 100$$

#### Total Nitrogen and Carbon

A sample of 0.5 g of freeze-dried and powdered material per replicate was used for N and C determination in fruit. The analysis was based on a complete combustion of the sample at 950 °C in the presence of oxygen, using a TruSpec CN elemental analyzer (Leco, MI, United States) (Gazulla et al., 2012).

### Data Analysis

A bifactorial (genotype and N treatment) analysis of variance was performed for every trait studied for the evaluation of differences among the accessions (genotypes, G), between N treatments (N), and for the occurrence of G × N interactions (Gomez and Gomez, 1984). In the ANOVA, two levels were established for the N treatment factor, corresponding to HN and LN, whereas levels of the genotype factor were the 44 accessions of the collection. Data from the six replicates per accession were used, making a total number of cases, *n* = 264. The block effect due to the experimental design was removed to evaluate the effect of the two factors and the interaction (Gomez and Gomez, 1984). Mean values and range for all traits were calculated from accession means for both LN (*n* = 44) and HN (*n* = 44). Comparisons of average differences between the sets of local (*n* = 39) and commercial (*n* = 5) varieties were assessed with a *t*-student test at *p* < 0.05 using R statistical software v4.0.2 (R Core Team, 2013). Only traits with significant differences between both groups in the two environments were considered as displaying a stable significant difference.

A principal component analysis (PCA) was performed using pairwise Euclidean distances among accession means for each N treatment and for all the traits. PCA loading and score plots were drawn using R package *ggplot2* (Wickham, 2016). Prediction ellipses for LN and HN with a 95% level of confidence were added to the PCA score plot. Phenotypic and environmental correlations among traits were calculated from accession means and residuals, respectively, using the R packages *psych* (Revelle, 2018) and *corrplot* (Wei et al., 2017). Pearson linear coefficients of correlation (r) were calculated between pairs of traits, and significance of correlations was evaluated with the Bonferroni test at *p* < 0.05 (Hochberg, 1988).

#### Genetic Parameters

The genotypic (σ^2^_*G*_) and phenotypic variances (σ^2^_*P*_ = σ^2^_*G*_ + σ^2^_*N*_ + σ^2^_*G* × *N*_) of each trait were obtained from the mean squares (MS) of the genotype, G × N interaction, and residuals of the ANOVA performed, in order to estimate broad-sense heritability (H^2^) using the formula H^2^ = σ_*G*_^2^/σ_*P*_^2^ (Wricke and Weber, 2010). Standard errors (SE) of the heritabilities were calculated by the Delta method, using the following formulas (Nyquist, 1991):

$$\text{SE}\left({\text{H}}^{2}\right)={\text{H}}^{2}\times \sqrt{\frac{\sigma_{\text{a}}^{2}}{{\text{A}}^{2}}+\frac{\sigma_{\text{b}}^{2}}{{\text{B}}^{2}}-2\times \frac{\text{cov}\left(A,B\right)}{\text{A}\times \text{B}}}$$

$$\text{A}=\frac{{\text{M}}_{1}-{\text{M}}_{2}}{\delta \times \text{r}}$$

$$\text{B}=\frac{{\text{M}}_{1}-{\text{M}}_{2}}{\delta \times \text{r}}+\frac{{\text{M}}_{2}-{\text{M}}_{3}}{\text{r}}+{\text{M}}_{3}$$

$$\sigma_{\text{a}}^{2}={\left(\frac{1}{\delta \times \text{r}}\right)}^{2}\times \left[\frac{2\times {\text{M}}_{1}^{2}}{{\text{df}}_{\mathrm{M1}}+2}+\frac{2\times {\text{M}}_{2}^{2}}{{\text{df}}_{\mathrm{M2}}+2}\right]$$

$$\sigma_{\text{b}}^{2}={\left(\frac{1}{\delta \times \text{r}}\right)}^{2}\times \frac{2\times {\text{M}}_{1}^{2}}{{\text{df}}_{\mathrm{M1}}+2}+\left(\frac{\delta -1}{\delta \times \text{r}}\right)$$

$$\;\times \frac{2\times {\text{M}}_{2}^{2}}{{\text{df}}_{\mathrm{M2}}+2}+{\left(1-\frac{1}{\text{r}}\right)}^{2}\times \frac{2\times {\text{M}}_{3}^{2}}{{\text{df}}_{\mathrm{M3}}+2}$$

where M_1_, M_2_, and M_3_ are the MS for genotype, G × N interaction, and residuals, respectively; df_*M1*_, df_*M2*_, and df_*M3*_ are the degrees of freedom on which M_1_, M_2_, and M_3_ were calculated, respectively, δ is the number of treatments, and r is the number of replications.

The coefficients of genetic (CV_*G*_) and phenotypic (CV_*P*_) variation were estimated from the corresponding variance components (σ^2^_*G*_ and σ^2^_*P*_) and the mean value of the trait (μ) as (Wricke and Weber, 2010)

$$\text{CV}=\frac{\sqrt{\sigma^{2}}}{\mu }\times 100$$

and their SE were calculated as

$$\text{SE}\left(\text{CV}\right)=\frac{\text{CV}\times \sqrt{1+2\times {\left(\mathrm{CV}/100\right)}^{2}}}{\sqrt{2\times \text{N}}}$$

where N is the total number of individuals used in the CV estimation.

## Results

### Analysis of Variance

Analysis of variance (ANOVA) revealed a significant effect of the genotype for all traits. For N treatment, no significant effect was observed, except for hue; contents of fructose, glucose, and total sugars; and total sweetness index (Table 2). For these five traits, F-ratio values were much greater than those of the genotype factor. However, no significant effect of the interaction G × N was detected for any of those traits. On the other hand, a significant G × N interaction was observed for lightness of color, fructose:glucose ratio, contents in lycopene and β-carotene, total carotenoids, and total carbon (Table 2). Except for lycopene and total carotenoid content, F-ratio values of the G × N interaction were lower than those of the genotype effect. In the cases of traits with a significant interaction G × N, it is worth mentioning the particular accessions that showed a significant difference between HN and LN and how the latter affected those traits. Therefore, for lightness of color, only three out of 44 accessions showed a significantly increased mean value under LN (MO1, MO2, TE1), among which MO2 had the largest increase (20%). For the fructose:glucose ratio, the same effect under LN was observed on three accessions (MO2, SN2, TO1), while AC1 and FA2 showed a significantly lower average fructose:glucose ratio. SN2 was the accession with the greatest difference between N treatments for the fructose:glucose ratio, which showed an increased mean value by 36% under LN. In the case of lycopene content, as well as for total carotenoids, LN significantly increased the mean values of three accessions (BL1, MT1, TO1), while it had the opposite effect on six accessions (AY1, LA2, LL2, TA1, VH2, VI1). For both lycopene and total carotenoid content, the largest difference between N treatments was found for BL1 (63% and 60% of increase under LN, respectively). Likewise, AC1, BL1, MT1, and TO1 showed a higher average β-carotene content under LN, and FI1, LA2, and VI1 had lower mean values of this trait under the same treatment. TO1 had the greatest percentage of increase for average β-carotene content under LN (45%), while VI1 had the highest percentage of decrease under the same conditions (47%). Finally, total carbon content was, in average, significantly higher under LN in four out of the 44 accessions evaluated (C4, C5, CO1, TO1). In this case, the commercial variety C5 had the highest percentage of increase (5%). Data of average values per accession and N treatment for each trait analyzed are included as Supplementary Table 3. For none of the traits where the G × N interaction was significant, a significant effect of N treatment was observed (Table 2).

**TABLE 2:** F-ratio values for genotype (G), nitrogen treatment (N; low or high), and genotype per nitrogen treatment interaction (G × N) of each trait evaluated in the present study, obtained from the bifactorial ANOVA, which considered the 44 ‘de penjar’ accessions of the collection with three replicates per N treatment (*n* = 44 × 2 × 3 = 264).

**Traits**	**Genotype (G)**	**N treatment (N)**	**G × N interaction**
*Vegetative and plant traits*			
spad	4.15***	0.15	1.23
y	3.64***	0.66	0.83
*Fruit traits*			
frw	19.04***	2.13	0.82
L	4.16***	1.04	1.64*
chroma	5.80***	0.00	1.17
hue	4.70***	16.86*	1.11
Nf	3.25***	3.77	0.94
Cf	3.32***	4.87	1.54*
pH	2.27***	0.42	1.32
ssc	4.68***	0.90	0.91
ta	5.04***	0.02	0.91
cit	5.37***	0.54	1.28
mal	10.44***	0.96	1.33
tacid	4.51***	0.45	1.33
citmalr	11.31***	1.40	1.10
fru	3.83***	53.25**	1.25
glu	4.40***	10.90*	1.35
tsug	4.19***	23.70**	1.27
fruglur	3.93***	1.39	2.19***
tsi	4.09***	32.16**	1.25
ssctar	4.64***	0.01	0.93
tsugtacidr	3.78***	2.40	0.88
tsitar	4.83***	1.19	0.96
vitc	2.39***	0.91	0.91
lyc	1.80**	0.40	2.23***
bcar	2.69***	2.40	2.04***
tcar	1.92**	0.71	2.25***
gluta	2.29***	0.23	1.22
aspa	2.66***	0.78	1.03

The full name of each trait abbreviation in the first column can be found in Table 1. Significant differences are indicated with * (*p* < 0.05), ** (*p* < 0.01), or *** (*p* < 0.001).

Mean values with their standard difference and range, as well as the paired difference between mean values in high N and low N treatment, of the whole ‘de penjar’ tomato collection (*n* = 44) under each N treatment, are shown in Table 3. Average yield and fruit weight were, respectively, 2.20 kg plant^–1^ and 72.7 g (LN) and 1.98 kg plant^–1^ and 70.4 g (HN), while for total N and C, mean values were 21.1 g kg^–1^ of dry matter (dm) and 408.0 g kg^–1^ dm (LN), and 19.9 g kg^–1^ dm and 405.8 g kg^–1^ dm (HN), respectively (Table 3). Average soluble solid content was 5.52% (LN) and 5.69% (HN), while pH and titratable acidity mean values were 4.13 and 0.57% (LN) and 4.11 and 0.58% (HN), respectively. Average values for total organic acids and reducing sugars were, respectively, 8.03 g kg^–1^ of fresh weight (fw) and 34.30 g kg^–1^ fw (LN) and 7.62 g kg^–1^ fw and 39.65 g kg^–1^ fw (HN). Mean values of the fructose:glucose ratio were slightly above 1.0 under both treatments (1.26 for LN and 1.19 for HN), while the citric:malic acid ratio was over 5.0. Regarding ratios between sugar and acid parameters (soluble solids content:titratable acidity, total sugars:total acids, and total sweetness index:titratable acidity), mean values were a great deal above 1.0 in both treatments with values of 10.54, 4.64, and 7.68, respectively, for LN and 10.63, 5.57, and 8.63, respectively, for HN (Table 3).

**TABLE 3:** Mean ± standard deviation (SD) and range, based on the accession averages, for the traits measured in the ‘de penjar’ tomato collection used in the present study in low- (LN) and high-nitrogen (HN) treatment (*n* = 44 × 2 = 88), and mean of the paired difference HN – LN, based on the accession averages (*n* = 44).

	**Low nitrogen (*n* = 44)**		**High nitrogen (*n* = 44)**		**Paired HN – LN (*n* = 44)**	
**Traits**	**Mean ± SD**	**Range**	**Mean ± SD**	**Range**	**Mean**
*Vegetative and plant traits*					
spad	49.64 ± 3.54	40.67–56.25	48.98 ± 5.41	36.38–60.90	–0.67
y (kg plant^–1^)	2.20 ± 0.47	0.96–3.18	1.98 ± 0.46	1.23–3.90	–0.22
*Fruit traits*					
frw (g)	72.7 ± 19.9	29.0–132.5	70.4 ± 20.3	29.3–134.6	–2.3
L	36.57 ± 2.95	32.01–43.29	35.50 ± 2.09	31.23–40.42	–1.07
chroma	21.50 ± 3.45	14.69–32.28	21.58 ± 3.28	15.30–33.23	0.08
hue (°)	40.00 ± 5.91	30.53–51.32	37.78 ± 4.19	29.27–46.11	−2.22*
Nf (g kg^–1^ dm)	21.1 ± 1.9	17.2–25.4	19.9 ± 2.2	14.6–26.6	–1.2
Cf (g kg^–1^ dm)	408.0 ± 3.1	402.3–416.3	405.8 ± 4.6	390.5–416.7	–2.26
pH	4.13 ± 0.17	3.83–4.63	4.11 ± 0.18	3.83–4.50	–0.02
ssc (%)	5.52 ± 0.57	4.28–6.58	5.69 ± 0.70	4.02–6.87	0.16
ta (%)	0.57 ± 0.13	0.34–0.96	0.58 ± 0.12	0.37–0.85	0.01
cit (g kg^–1^ fw)	6.73 ± 1.77	4.22–12.28	6.30 ± 1.62	4.31–12.66	–0.43
mal (g kg^–1^ fw)	1.30 ± 0.36	0.73–2.00	1.33 ± 0.33	0.83–2.36	0.03
tacid (g kg^–1^ fw)	8.03 ± 1.78	5.22–13.60	7.62 ± 1.60	5.55–13.98	–0.41
citmalr	5.71 ± 2.25	2.69–10.02	5.14 ± 1.99	2.75–9.44	–0.57
fru (g kg^–1^ fw)	18.83 ± 2.41	13.69–22.77	21.28 ± 2.46	16.31–27.50	2.45**
glu (g kg^–1^ fw)	15.47 ± 3.02	9.57–21.52	18.37 ± 3.06	10.99–24.93	2.90*
tsug (g kg^–1^ fw)	34.30 ± 5.17	24.27–42.87	39.65 ± 5.29	28.96–52.07	5.35**
fruglur	1.26 ± 0.18	1.01–1.88	1.19 ± 0.15	1.02–1.93	–0.06
tsi	4.00 ± 0.56	2.86–4.94	4.59 ± 0.58	3.43–5.97	0.59**
ssctar	10.54 ± 2.65	5.74–19.27	10.63 ± 2.38	6.84–16.41	0.09
tsugtacidr	4.64 ± 1.21	2.29–7.79	5.57 ± 1.15	3.11–8.70	0.92
tsitar	7.68 ± 2.14	4.14–14.40	8.63 ± 2.10	5.21–13.65	0.95
vitc (g kg^–1^ fw)	0.27 ± 0.04	0.15–0.39	0.29 ± 0.04	0.21–0.38	0.02
lyc (mg kg^–1^ fw)	7.99 ± 2.22	3.64–15.87	7.64 ± 2.84	3.34–14.50	–0.34
bcar (mg kg^–1^ fw)	3.45 ± 0.84	2.00–5.72	3.17 ± 0.80	1.96–5.15	–0.28
tcar (mg kg^–1^ fw)	15.41 ± 4.15	7.51–29.71	14.58 ± 5.04	6.79–26.01	–0.83
gluta (g kg^–1^ fw)	3.42 ± 1.08	1.73–7.01	3.83 ± 0.78	2.34–5.61	0.41
aspa (g kg^–1^ fw)	0.76 ± 0.21	0.33–1.33	0.90 ± 0.19	0.51–1.40	0.14

The full name of each trait abbreviation in the first column is found in Table 1. Significant differences between LN and HN treatments are based on the results of the ANOVA (*n* = 264) and indicated with * (*p* < 0.05) and ** (*p* < 0.01).

When comparing significantly different average values between the N treatments, taking high N conditions as a reference, hue was significantly higher (5.6%) under low N conditions, while it was significantly lower for contents in fructose (−11.5%), glucose (−15.8%), total sugars (−13.5%), and total sweetness index (−12.9%).

**FIGURE 1:**
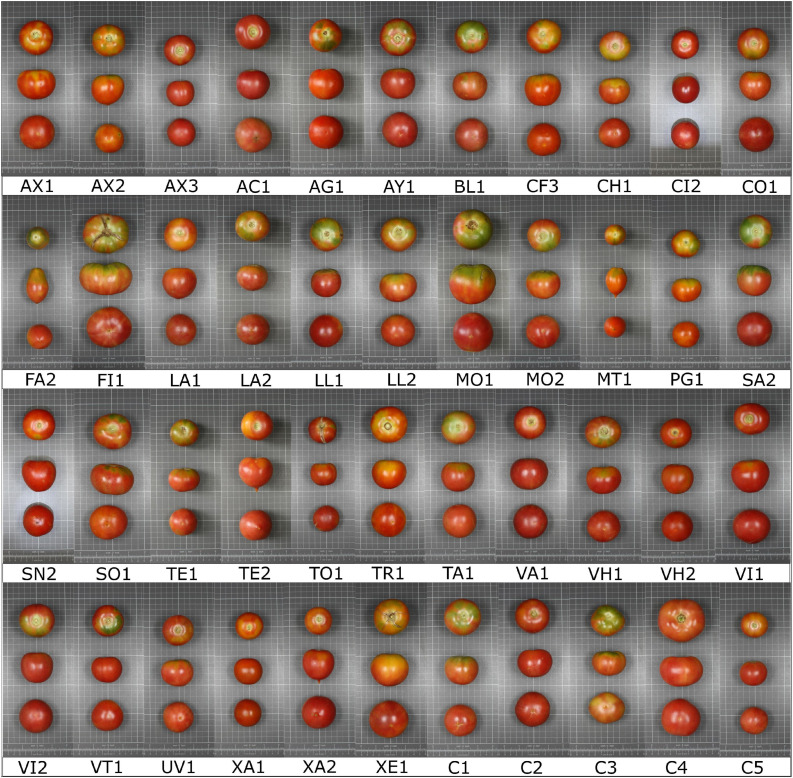
Representative fruits of the 39 ‘de penjar’ tomato local varieties and the five commercial varieties (accession name for each code is found in Supplementary Table 1) used for agronomic and composition characterization under two levels of nitrogen inputs. The grid cells in the pictures measure 1 × 1 cm.

Considerable variation was found among the 44 varieties under both conditions for the traits evaluated. Representative fruits of the ‘de penjar’ collection studied are pictured in Figure 1. In this way, the traits with a larger value for the relative range of variation (maximum/minimum values) for LN conditions were fruit weight (4.57-fold), lycopene content (4.36-fold), and glutamic acid content (4.05-fold). Under HN, fruit weight (4.59-fold) and lycopene content (4.34-fold) were also the traits with the highest relative range of variation, followed by total carotenoid content (3.83-fold). On the other side, the traits with the lowest values for the relative range of variation were total C content (1.04-fold for LN and 1.07-fold for HN), pH (1.21-fold for LN and 1.18-fold for HN), and lightness of color (1.35-fold for LN and 1.29-fold for HN). For all traits, the ranges of variation between both N conditions based on the accession means overlapped to a large extent (Table 3).

### Differences Between Varietal Types

The groups of local varieties (*n* = 39) on one side and commercial varieties (*n* = 5) on the other did not display significant differences (*p* < 0.05) between them under both LN and HN conditions for most of the traits analyzed (Table 4). However, significant differences were found for ratios of both soluble solid content and total sweetness index with titratable acidity, and vitamin C content. For each of these traits, local varieties presented higher means than commercial varieties in both conditions. Thus, the average ratio between soluble solid content and titratable acidity for local varieties was 18.4% higher under LN and 30.0% under HN. The same occurred for the average ratio between total sweetness index and titratable acidity, with values being 22.0% (LN) and 28.8% (HN) higher in local varieties over commercial ones, as well as for vitamin C content, with the former displaying higher mean values by 25.0% (LN) and 17.2% (HN) (Table 4).

**TABLE 4:** Mean ± standard error (SE) and *t* value of the traits that showed a significant difference between local (L; *n* = 39) and commercial (C; *n* = 5) varieties used in the present study, for both nitrogen treatments.

	**Low nitrogen**			**High nitrogen**		
	**Mean ± SE**		***t* value**	**Mean ± SE**		***t* value**
**Traits**	**L (*n = 39)***	**C (*n* = 5)**		**L (*n* = 39)**	**C (*n* = 5)**	
ssctar	10.76 ± 0.44	8.78 ± 0.25	3.96***	11.00 ± 0.36	7.70 ± 0.26	7.37***
tsitar	7.87 ± 0.35	6.14 ± 0.48	2.92*	8.92 ± 0.33	6.35 ± 0.24	6.36***
vitc (g kg^–1^ fw)	0.28 ± 0.01	0.21 ± 0.02	2.78*	0.29 ± 0.01	0.24 ± 0.02	3.06*

The full name of each trait abbreviation in the first column is found in Table 1. Significant differences between varietal type are indicated with * (*p* < 0.05), ** (*p* < 0.01), *** (*p* < 0.001).

### Genetic Parameters

Heritability in a broad sense (H^2^), genetic and phenotypic coefficients of variation (CV_*G*_ and CV_*P*_) for the traits studied are presented in Table 5. Considering both treatments and their interaction, fruit weight had the greatest H^2^ with 0.76. Apart from that, only malic acid and citric:malic ratio showed a broad-sense heritability higher than 0.50. On the other hand, lycopene, β-carotene, and total carotenoid content had the lowest H^2^. As expected, the coefficient of phenotypic variance was higher than the coefficient of genetic variance for all traits studied. The lowest percentages of CV_*G*_ were obtained for lycopene and total carotenoids, which, at the same time, had the highest values of CV_*P*_, together with citric:malic acid ratio. The greatest values of CV_*G*_ were found for citric:malic acid ratio, fruit weight, and malic acid content. Finally, carbon content in fruit, pH, and lightness of color had the lowest values of CV_*P*_.

**TABLE 5:** Broad sense heritability estimates (H^2^), genotypic and phenotypic variance coefficient (CV_*G*_ and CV_*P*_, respectively), and their standard errors of the traits analyzed in our ‘de penjar’ tomato collection taking into account the two nitrogen conditions.

**Traits**	**H^2^ ± SE**	**CV_*G*_ (%) ± SE**	**CV_*P*_ (%) ± SE**
*Vegetative and plant traits*			
spad	0.31 ± 0.08	6.82 ± 0.73	12.23 ± 1.32
y	0.33 ± 0.07	17.70 ± 1.95	30.77 ± 3.58
*Fruit traits*			
frw	0.76 ± 0.04	26.90 ± 3.07	30.78 ± 3.58
L	0.26 ± 0.08	4.68 ± 0.50	9.23 ± 0.99
chroma	0.42 ± 0.07	12.74 ± 1.38	19.60 ± 2.17
hue	0.37 ± 0.07	10.36 ± 1.12	17.12 ± 1.88
Nf	0.30 ± 0.07	7.51 ± 0.80	14.14 ± 1.54
Cf	0.40 ± 0.15	0.58 ± 0.06	1.29 ± 0.14
pH	0.12 ± 0.07	2.22 ± 0.24	6.28 ± 0.67
ssc	0.39 ± 0.07	9.36 ± 1.01	14.94 ± 1.63
ta	0.41 ± 0.07	18.21 ± 2.00	28.27 ± 3.25
cit	0.38 ± 0.08	20.44 ± 2.27	32.99 ± 3.88
mal	0.58 ± 0.07	23.19 ± 2.60	30.52 ± 3.54
tacid	0.32 ± 0.08	15.96 ± 1.74	28.08 ± 3.22
citmalr	0.62 ± 0.06	35.53 ± 4.24	45.05 ± 5.69
fru	0.28 ± 0.07	8.60 ± 0.92	16.13 ± 1.76
glu	0.31 ± 0.08	13.09 ± 1.42	23.39 ± 2.63
tsug	0.31 ± 0.08	10.36 ± 1.12	18.64 ± 2.05
fruglur	0.17 ± 0.09	7.24 ± 0.78	17.46 ± 1.92
tsi	0.28 ± 0.07	9.69 ± 1.04	17.59 ± 1.93
ssctar	0.39 ± 0.07	19.43 ± 2.15	31.20 ± 3.64
tsugtacidr	0.33 ± 0.07	18.24 ± 2.01	31.54 ± 3.68
tsitar	0.20 ± 0.04	21.29 ± 2.37	33.85 ± 4.00
vitc	0.200.06	9.481.02	21.102.35
lyc	0.00 ± 0.07	0.00 ± 0.00	46.20 ± 5.88
bcar	0.07 ± 0.08	9.05 ± 0.97	33.22 ± 3.91
tcar	0.00 ± 0.08	0.00 ± 0.00	43.08 ± 5.38
gluta	0.14 ± 0.07	14.34 ± 1.56	37.97 ± 4.59
aspa	0.21 ± 0.07	16.26 ± 1.78	35.31 ± 4.21

*The full name of each trait abbreviation in the first column is found in Table 1.*

### Correlation Among Traits

**FIGURE 2:**
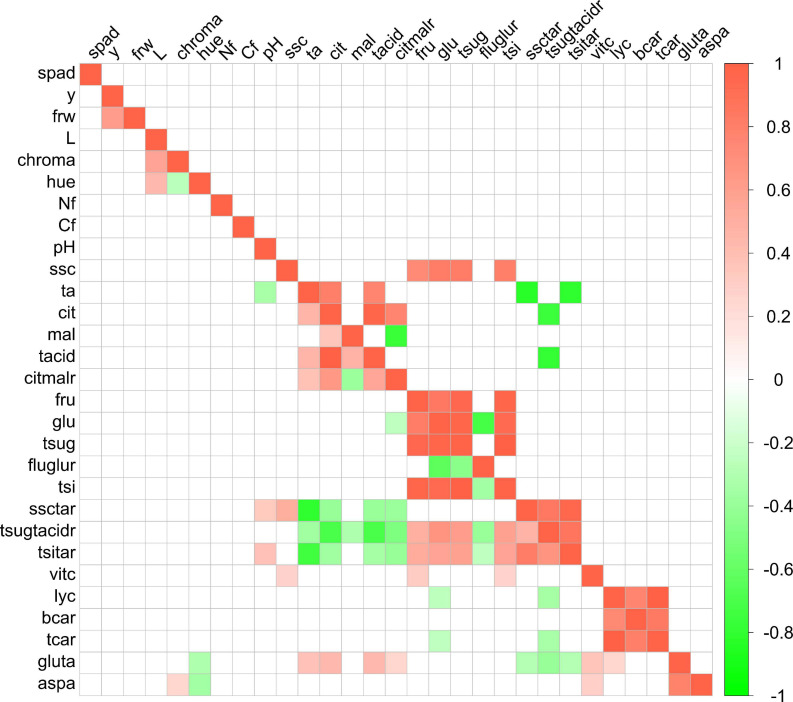
Correlogram among traits evaluated in the ‘de penjar’ tomato collection. Phenotypic correlations are shown above the diagonal; environmental correlations below the diagonal. Only significant correlations at *p* < 0.05 according to the Bonferroni test are displayed. Green and red colors correspond to negative and positive correlations, respectively. The full name of each trait abbreviation is found in Table 1.

Few significant (*p* < 0.05) phenotypic correlations were detected (Figure 2). In addition, the analysis did not reveal any significant correlation between SPAD, yield, or fruit weight on one side and fruit composition traits on the other. As for phenotypic correlations, fructose and glucose contents were positively and significantly correlated with each other (*r* = 0.84) and with total sugar content (*r* = 0.95 and 0.97, respectively), with total sweetness index (*r* = 0.98 and 0.94, respectively), and, with lower correlation coefficient values, with soluble solid content (*r* = 0.73 and 0.82, respectively). The same pattern was observed for correlations between citric acid content and citric:malic ratio (*r* = 0.77), total acid content (*r* = 0.98), or titratable acidity (*r* = 0.80). Citric acid content was also negatively correlated with total sugars:total acids ratio (*r* = −0.75). On the contrary, malic acid content was not significantly correlated with any of these traits. Different carotenoid contents were positively correlated with each other, with *r* = 0.76. Lycopene displayed higher correlation coefficient values than β-carotene to total carotenoids (*r* = 0.99 and 0.84, respectively) (Figure 2).

In the case of environmental correlations, the number of significant ones was higher than for phenotypic correlations (Figure 2). The same strong correlations found among traits in the phenotypic analysis were detected. Fruit weight and yield were environmentally correlated with *r* = 0.61. In the same way, a significant but slightly lower correlation was detected between vitamin C and traits related to sugar content, with r values between 0.26 and 0.32. Vitamin C also had a significant and positive environmental correlation to aspartic and glutamic acid contents (*r* = 0.29 and 0.37, respectively). With respect to these amino acids, they were environmentally intercorrelated, with *r* = 0.79. Aspartic acid also presented a negative significant correlation to color parameters chroma (*r* = 0.26) and hue (*r* = −0.35), and positive, to vitamin C content (*r* = 0.29). Glutamic acid displayed significant positive environmental correlations to acidity and related traits, with r between 0.25 and 0.44, to vitamin C (*r* = 0.37) and lycopene contents (*r* = 0.24), but negative significant correlations to sugar:acid ratio-related traits (*r* = −0.28 to −0.40). Glucose content and total sugar:total acid ratio also showed negative environmental correlations to lycopene (*r* = −0.25 and −0.35, respectively) and total carotenoid content (*r* = −0.25 and −0.34, respectively) (Figure 2).

### Principal Component Analysis

**FIGURE 3:**
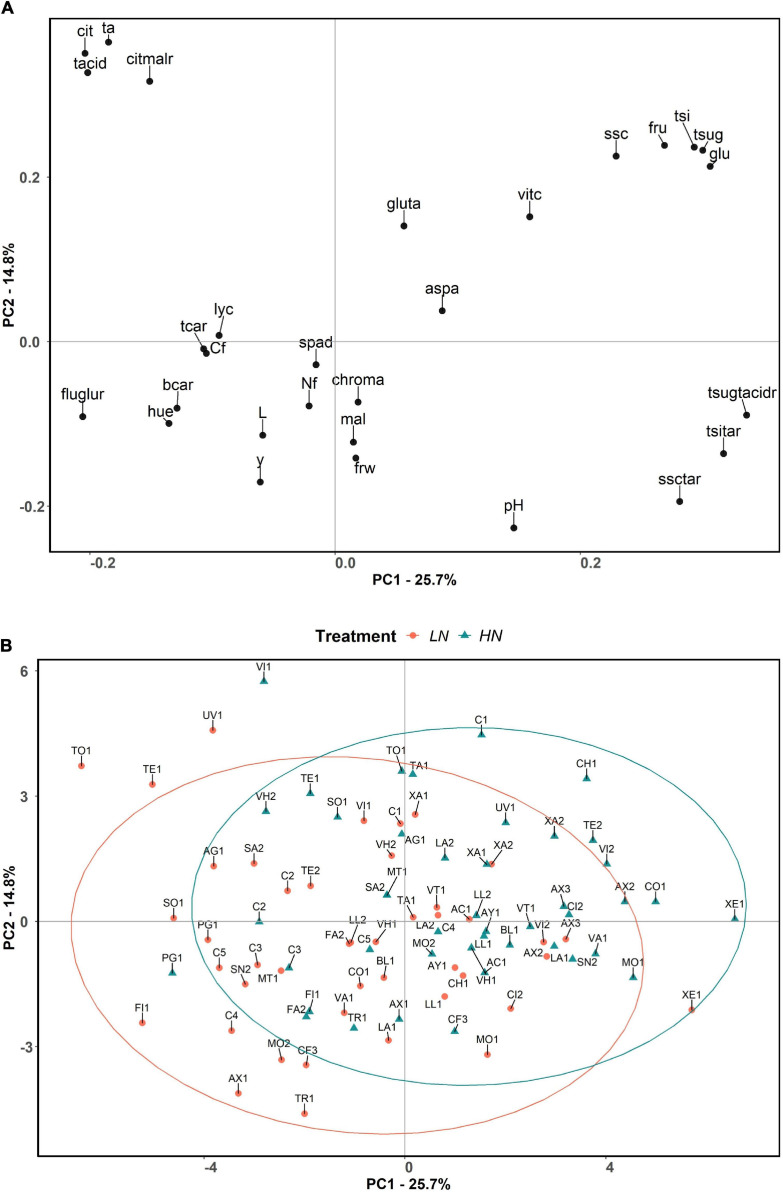
Principal component analysis loading plot **(A)** and score plot **(B)** evaluated in the present study, based on the two first principal components of PCA. The first and second components account for 25.7 and 14.8% of the total variation, respectively. The accessions are represented by different symbols and color according to the treatment in which they were grown: orange colored circle for low nitrogen, and blue colored triangle for high nitrogen. Ellipses grouped the accessions for each treatment with a 95% confidence level. The full name of each trait abbreviation is found in Table 1. Accession name for each code can be found in Supplementary Table 1.

The first two principal components (PCs) of the PCA explained 40.5% of the total variation observed, with PC1 and PC2 accounting for 25.7% and 14.8% of the total variation, respectively. Total sugar:total acid ratio, total sweetness index:titratable acidity ratio, and glucose content were the traits displaying the highest positive correlation with PC1, while fructose:glucose ratio, citric acid, and total acid contents were the ones with the highest absolute negative values with PC1. On the other hand, titratable acidity and citric acid and total acid contents also displayed high positive correlations with PC2, whereas pH, soluble solid content:titratable acidity ratio, and yield were highly negatively correlated with PC2 (Figure 3).

The principal component analysis did not clearly separate the HN and LN treatments, although for the same variety, compared to the control, the projection corresponding to the LN treatment tended to display lower values for PC1 and PC2, which is associated with higher content of carotenoids, fructose:glucose ratio, hue, and slightly higher fruit carbon and nitrogen contents and yield, and associated with lower contents of sugars and slightly lower vitamin C (Figure 3).

It is noteworthy that a group of accessions under HN conditions (CH1, UV1, XA2, TE2, AX2, CO1, VI2, C1) plotted in the second quadrant of the PCA (positive values for PC1 and PC2), being associated with higher total sweetness index, total and individual contents of sugars, soluble solids and their ratios with total acids and titratable acidity, and vitamin C and glutamic and aspartic acid contents. Another group to highlight is that of the accessions AX1, FI1, TR1, C4, MO2, and CF3 under LN conditions, which plotted in the third quadrant (negative values of PC1 and PC2), which are associated with brighter fruits with external color tending to orange, higher fructose:glucose ratio, and β-carotene content, in addition to high yield. Besides that, there are some accessions that appear outside the 95% confidence ellipses. In this respect, UV1, TE1, and TO1 under LN conditions and VI1 under HN produced the most acidic tomatoes. XE1 under LN conditions also remained outside the ellipse, presenting high sugar contents, resulting in greater ratios of total sugars:total acids, soluble solid content:titratable acidity, and total sweetness index:titratable acidity. Finally, some accessions (AX3, AC1, TR1, XA1, XA2, XE1, and C3) seemed to perform similarly under LN and HN conditions as they appeared very close to each other according to PC1 and PC2 (Figure 3).

### Characteristics of Highest-Yielding Varieties

Table 6 shows the ranking of the 10 best ‘de penjar’ tomato accessions evaluated in the present work for yield, with their performance and ranking position within the entire collection for the most important composition traits, under both treatments separately. The ranking of the top 10 ‘de penjar’ tomato accessions of the collection for resilience in yield, with their resilience index and ranking position within the entire collection for the most important composition traits, is displayed in Table 7.

**TABLE 6:** Ranking of the 10 best ‘de penjar’ tomato accessions evaluated in the present work for yield, with their performance and ranking position (in parentheses) within the entire collection for the most important composition traits, in both treatments separately.

	**y (kg plant^–1^)**	**frw (g)**	**Nf (g kg^–1^ dm)**	**tsug (g kg^–1^ fw)**	**fruglur**	**tacid (g kg^–1^ fw)**	**citmalr**	**tsugtacidr**	**vitc (g kg^–1^ fw)**	**tcar (mg kg−^1^ fw)**	**bcar (mg kg−^1^ fw)**	**gluta (g kg−^1^ fw)**	**aspa (g kg−^1^ fw)**
*Low nitrogen*													
TR1	3.18 (1)	84.5 (10)	20.4 (31)	29.32 (36)	1.24 (22)	6.09 (40)	3.58 (37)	5.05 (17)	0.24 (38)	16.88 (14)	4.59 (4)	2.08 (41)	0.56 (36)
MT1	3.13 (2)	29.0 (44)	19.8 (33)	37.09 (15)	1.25 (20)	7.38 (30)	3.30 (40)	5.14 (14)	0.25 (29)	25.39 (2)	5.72 (1)	2.95 (28)	0.58 (34)
FI1	3.06 (3)	132.5 (1)	20.7 (28)	25.66 (41)	1.50 (4)	10.32 (4)	4.24 (27)	2.49 (43)	0.29 (16)	14.36 (28)	3.33 (27)	2.54 (36)	0.33 (44)
MO2	2.90 (4)	86.5 (7)	18.2 (42)	31.35 (33)	1.61 (3)	7.41 (28)	3.17 (41)	4.31 (28)	0.27 (18)	17.96 (10)	3.82 (14)	3.24 (22)	0.71 (26)
C2	2.89 (5)	83.7 (12)	19.4 (35)	34.22 (24)	1.19 (26)	8.85 (11)	7.36 (13)	3.97 (32)	0.24 (32)	14.37 (27)	3.41 (24)	2.33 (38)	0.64 (30)
C1	2.65 (6)	85.1 (8)	20.4 (30)	42.68 (3)	1.14 (32)	8.49 (15)	7.53 (12)	5.07 (15)	0.24 (36)	15.52 (18)	3.56 (19)	3.98 (12)	0.89 (9)
MO1	2.62 (7)	117.8 (2)	22.9 (8)	35.47 (17)	1.01 (44)	5.73 (42)	6.07 (20)	6.20 (6)	0.25 (30)	12.58 (34)	3.44 (23)	3.53 (15)	0.75 (23)
CO1	2.62 (8)	76.7 (20)	21.1 (23)	33.79 (25)	1.10 (38)	6.87 (34)	6.14 (19)	5.03 (18)	0.24 (39)	12.64 (33)	2.62 (36)	2.87 (30)	0.85 (15)
C3	2.59 (9)	64.8 (29)	18.2 (41)	32.49 (30)	1.38 (7)	10.26 (5)	4.67 (22)	3.32 (37)	0.26 (28)	14.08 (32)	4.08 (8)	2.32 (39)	0.50 (40)
AX1	2.58 (10)	83.2 (13)	23.9 (3)	24.27 (44)	1.31 (12)	6.47 (36)	4.14 (32)	4.06 (31)	0.23 (40)	17.42 (12)	3.90 (13)	3.50 (16)	0.89 (10)
*High nitrogen*													
FI1	3.90 (1)	134.6 (1)	18.6 (33)	33.91 (37)	1.33 (5)	8.41 (12)	4.66 (20)	4.41 (37)	0.29 (24)	19.54 (8)	5.15 (1)	3.81 (19)	0.78 (33)
MO1	2.89 (2)	126.9 (2)	20.0 (21)	42.80 (12)	1.13 (29)	7.37 (20)	4.11 (25)	6.25 (12)	0.38 (1)	15.94 (17)	3.59 (16)	3.73 (25)	0.81 (30)
AX1	2.77 (3)	102.2 (3)	22.5 (3)	33.23 (38)	1.21 (18)	5.95 (40)	3.84 (27)	5.73 (21)	0.29 (22)	12.77 (28)	2.86 (28)	3.80 (20)	1.00 (12)
TR1	2.69 (4)	79.0 (12)	19.8 (23)	32.02 (40)	1.23 (13)	5.55 (44)	3.48 (34)	5.84 (18)	0.27 (32)	15.72 (18)	3.69 (14)	2.40 (43)	0.60 (41)
C3	2.50 (5)	59.1 (34)	14.6 (44)	31.99 (41)	1.31 (6)	7.94 (14)	4.18 (23)	4.29 (39)	0.21 (43)	11.29 (31)	2.97 (24)	2.43 (42)	0.58 (42)
MT1	2.48 (6)	29.3 (44)	16.6 (42)	38.86 (25)	1.18 (19)	7.03 (26)	4.34 (22)	5.57 (22)	0.21 (44)	12.48 (29)	3.24 (18)	4.71 (6)	0.93 (20)
XE1	2.35 (7)	97.7 (5)	21.2 (11)	52.07 (1)	1.08 (35)	5.93 (41)	3.66 (31)	8.70 (1)	0.33 (5)	17.56 (12)	3.88 (11)	3.74 (24)	0.84 (24)
C4	2.24 (8)	100.0 (4)	26.6 (1)	37.97 (27)	1.14 (26)	6.69 (31)	3.99 (26)	5.90 (17)	0.25 (38)	11.76 (30)	2.87 (27)	5.16 (3)	1.30 (2)
CF3	2.21 (9)	84.8 (8)	19.7 (24)	36.49 (35)	1.21 (16)	5.86 (42)	3.34 (37)	6.30 (11)	0.27 (34)	19.60 (7)	4.05 (6)	3.43 (29)	0.75 (36)
AC1	2.19 (10)	80.2 (11)	19.3 (29)	36.92 (32)	1.50 (2)	6.09 (39)	5.08 (19)	6.08 (14)	0.27 (33)	17.14 (13)	3.10 (21)	3.93 (18)	1.10 (6)

*The full name of each trait abbreviation in the first row is found in Table 1.*

**TABLE 7:** Ranking of the 10 best ‘de penjar’ tomato accessions evaluated in the present work for resilience in yield, calculated as the ratio between mean value under low N treatment and mean value under high N treatment. Resilience and ranking position (in parentheses) within the entire collection of these 10 varieties for the most important composition traits are also shown.

	**y**	**frw**	**Nf**	**tsug**	**fruglur**	**tacid**	**citmalr**	**tsugtacidr**	**vitc**	**tcar**	**bcar**	**gluta**	**aspa**
*Resilience index*													
XA1	1.79 (1)	1.02 (24)	1.17 (7)	1.00 (8)	1.01 (31)	1.34 (3)	1.54 (2)	0.72 (31)	0.91 (26)	0.71 (37)	0.75 (37)	1.25 (7)	1.13 (7)
C2	1.55 (2)	1.19 (5)	0.93 (42)	1.12 (2)	1.02 (28)	1.13 (16)	1.02 (30)	0.95 (15)	1.12 (4)	1.82 (7)	1.40 (10)	0.89 (22)	0.75 (31)
MO2	1.45 (3)	1.23 (3)	0.96 (37)	0.83 (25)	1.29 (3)	1.05 (25)	1.02 (31)	0.80 (26)	0.97 (20)	1.16 (22)	0.96 (29)	0.78 (31)	0.86 (21)
C1	1.44 (4)	1.27 (2)	1.16 (12)	0.94 (12)	1.09 (16)	0.94 (34)	1.09 (23)	0.98 (12)	0.81 (35)	1.82 (8)	1.53 (7)	0.92 (20)	0.90 (19)
PG1	1.36 (5)	0.88 (41)	0.95 (40)	1.09 (3)	1.01 (30)	0.98 (33)	0.98 (33)	1.06 (5)	1.07 (10)	0.77 (35)	0.95 (31)	0.81 (29)	0.81 (27)
SO1	1.32 (6)	1.17 (6)	0.94 (41)	0.72 (41)	1.14 (9)	0.99 (31)	1.12 (17)	0.76 (30)	0.92 (25)	1.70 (9)	1.13 (23)	0.88 (24)	0.95 (13)
VI2	1.32 (7)	1.10 (13)	1.06 (23)	0.92 (15)	1.09 (17)	1.00 (30)	1.29 (7)	0.86 (22)	0.82 (34)	1.07 (25)	1.16 (22)	0.50 (43)	0.44 (43)
FA2	1.29 (8)	1.03 (23)	1.00 (30)	1.20 (1)	0.65 (44)	1.17 (15)	1.03 (28)	1.11 (3)	1.12 (5)	1.05 (26)	0.94 (33)	0.59 (40)	0.65 (38)
VT1	1.29 (9)	1.11 (11)	1.12 (16)	1.03 (5)	0.95 (38)	1.25 (8)	0.79 (41)	0.81 (23)	0.87 (32)	1.19 (20)	1.33 (17)	1.06 (11)	0.81 (25)
CO1	1.29 (10)	1.19 (4)	1.04 (24)	0.74 (39)	0.95 (39)	1.06 (22)	1.07 (27)	0.71 (34)	0.77 (38)	1.56 (11)	1.20 (20)	0.85 (26)	0.80 (28)

*The full name of each trait abbreviation in the first row is found in Table 1.*

Several local varieties showed comparable or even better performance in yield and other composition traits than commercial varieties, both in LN and HN. As far as commercial varieties are concerned, in LN conditions, C2, C1, and C3 ranked fifth (2.89 kg plant^–1^), sixth (2.65 kg plant^–1^), and ninth (2.59 kg plant^–1^) in yield, while only C3 and C4 were among the 10 best-yielding varieties in HN with 2.50 kg plant^–1^ (5th place) and 2.24 kg plant^–1^ (8th place) (Table 6). It is worth noting that, in addition, C2 and C1 ranked second (1.55) and fourth (1.44), respectively, in terms of yield resilience (Table 7). Regarding local varieties, TR1, MT1, and FI1 stood out as the three best-yielding varieties in LN (3.18, 3.13, and 3.06 kg plant^–1^, respectively), while FI1, MO1, and AX1 were the highest yielding in HN (3.90, 2.89, and 2.77 kg plant^–1^, respectively) (Table 6). None of these accessions were among the top 10 in resilience, although MO2 and CO1, which appeared among the 10 best accessions for yield in LN, ranked third (1.45) and tenth (1.29), respectively, for yield resilience (Table 7). When considering the rest of traits, there is a wide variation among the two sets of 10 highest-yielding accessions. Among them, FI1 ranked first in both yield and fruit weight (134.6 g) in HN. Contrarily, MT1, with the lowest fruit mean weight in the whole collection (29.0 g in LN and 29.3 g in HN), appeared as the second and sixth best-yielding variety in LN (3.13 kg plant^–1^) and HN (2.48 kg plant^–1^), respectively (Table 6). Taking into account the average content of N in fruit, the local variety AX1 ranked third under both LN (23.9 g kg^–1^ dm) and HN (22.5 g kg^–1^ dm) (Table 6). Regarding sweetness, it is worth mentioning the local variety XE1, which presented an outstanding average in total sugar content (52.07 g kg^–1^ fw), ranking first, under HN. Furthermore, it also showed the highest average total sugar:total acid ratio under the same conditions (8.70). On the other hand, MO2, FI1, and C3 were among the top 10 varieties in fructose:glucose ratio under LN with mean values of 1.61, 1.50, and 1.38, respectively, while AC1 and again FI1 and C3 had the highest mean values in HN (1.50, 1.33, and 1.31, respectively) (Table 6). In general, the 10 best-yielding varieties, under LN and HN, ranked in intermediate or low positions for both average total acid content and citric:malic ratio (Table 6). Regarding main antioxidants evaluated, MO1 was the accession with the greatest average content of vitamin C (0.38 g kg^–1^ fw) under HN, followed by XE1 (5th place; 0.33 g kg^–1^ fw). MT1 stood out for accumulating, in average, the highest concentrations of β-carotene (5.72 mg kg^–1^ fw) and the second highest of total carotenoids (25.39 mg kg^–1^ fw) in their fruits, under LN, while under HN, FI1 ranked first in β-carotene content (5.15 mg kg^–1^ fw) (Table 6). As for glutamic and aspartic acid contents, any of the local varieties in both sets of best-yielding varieties ranked above commercial varieties C1 under LN and C4 under HN. However, MT1 was placed within the top 10 positions for glutamic acid under HN (6th; 4.71 g kg^–1^ fw). Regarding aspartic acid, the same happened for AX1 (10th; 0.89 g kg^–1^ fw) and AC1 (6th; 1.10 g kg^–1^ fw) under LN and HN, respectively (Table 6).

When considering the average resilience indexes of the other traits for the top 10 accessions in yield resilience, it is worth mentioning some accessions of both local and commercial varieties. As for local varieties, MO2 ranked third for fructose:glucose resilience (1.29); FA2 stood out for having the highest resilience in total sugar content (1.20) and ranked third for total sugar:total acid resilience (1.11); XA1, which ranked first for yield resilience (1.79), had a remarkable resilience in glutamic and aspartic acid (1.25 and 1.13, respectively), ranking seventh for both of them (Table 7). With respect to commercial varieties, C2 stood out for its resilience in vitamin C (1.12) and total carotenoid content (1.82), ranking fourth and seventh, respectively, while C1 was highlighted for its resilience in β-carotene content (1.53), ranking seventh in the collection (Table 7).

## Discussion

The present work constitutes the first study of ‘de penjar’ tomato involving different doses of N fertilization. A diverse array of 39 local varieties together with five commercial varieties were characterized in open field under two nitrogen fertilization levels, one corresponding to similar values of their traditional cultivation (162 kg ha^–1^; high N) (Seda and Muñoz, 2011) and the other to less than one third of the first dosage (49 kg ha^–1^; low N). The large variability existing among the ‘de penjar’ collection for morphological, agronomic, and quality traits observed in this study paves the way for selection and breeding of this overlooked type of tomato. In terms of yield, average production in our collection (around 2 kg plant^–1^) stands on intermediate values reported for other LSL varieties from the eastern Spain (‘de penjar’) and Balearic Islands (‘de ramellet’) (Casals et al., 2012; Cebolla-Cornejo et al., 2013; Figàs et al., 2015, 2018b; Fullana-Pericàs et al., 2019). Our data about fruit mean weight are in agreement with Conesa et al. (2020). Regarding fruit quality, soluble solid content in the collection studied ranged around 4–7%, with an average of 5.5%, similar to values found in Fullana-Pericàs et al. (2019) but slightly lower to the values found in Figàs et al. (2015, 2018b). Similarly, when looking at individual concentrations of reducing sugars, mean values of fructose and glucose under both low and high N treatment fitted within the range of values obtained in Casals et al. (2015), which evaluated the sugar and acid profile of 25 accessions of ‘de penjar’ tomato. However, the ranges obtained by Casals et al. (2015) were wider and showed higher maximum average values by 1.26- and 1.19-fold, respectively, than the collection of the present study. The average titratable acidity in the present collection was also similar to those found for other ‘de penjar’ and ‘de ramellet’ tomato varieties (Cebolla-Cornejo et al., 2013; Figàs et al., 2015, 2018b). However, none of the varieties showed means of titratable acidity higher than 1.0%, while this commonly happens in ‘de ramellet’ tomato (Fullana-Pericàs et al., 2019). This might be due to genetic differences or to differences in the ripening stage at the time of harvesting. In any case, our work supports previous information indicating that LSL varieties from Spain generally are slightly more acidic than the varieties from Italy, while the latter accumulate more soluble solids (Conesa et al., 2020). On the other side, as opposed to fructose and glucose contents, the ‘de penjar’ tomato collection of the present work showed mean values of individual citric and malic acid content slightly higher than in the collection evaluated in Casals et al. (2015), with larger ranges of variation among accessions. In addition, the maximum average values found in the present collection for contents in citric and malic acid were 12.66 g kg^–1^ fw and 2.36 g kg^–1^ fw, respectively, while the maximum values in Casals et al. (2015) were 1.78- and 1.50-fold lower, respectively. Ranges for content in glutamic acid in the ‘de penjar’ collection of this study showed wider variation than in other works (Casals et al., 2015), although both glutamic and aspartic acids are compounds still barely studied in ‘de penjar’ tomatoes.

Compared with USDA standard nutritional references of cultivated tomato (Haytowitz et al., 2011), the ‘de penjar’ collection studied contained in average around 1.5-fold more glucose and fructose and 2-fold more vitamin C. However, lycopene and β-carotene contents were 3.3- and 1.4-fold, respectively, lower in ‘de penjar’ fruits, probably due to the pleiotropic effect of the *alc* mutation (Kumar et al., 2018). The same trend was observed for glutamic and aspartic acids, with mean contents of 1.2- and 1.6-fold lower in our collection compared to the USDA standard nutritional references (Haytowitz et al., 2011). This confirms that ‘de penjar’ LSL varieties are very different in composition terms to standard tomato varieties (Cebolla-Cornejo et al., 2013; Figàs et al., 2015).

### Importance of Soil Conditions and Cultivation Practices

Some recent studies have addressed the impact of organic farming alone and together with low N inputs on agronomic and quality traits of tomato. De Pascale et al. (2016) suggested that organic cultivation practices might be a better approach than conventional methods for improving yield and nutritional quality of tomato under limiting N and water conditions, although it depends on cultivar and soil type. In this study, ‘de penjar’ tomato was cultivated following organic farming practices. In this respect, ‘de penjar’ tomato have been traditionally cultivated in open field under rain-fed, low-input conditions, which makes this tomato crop ideal for adaptation to organic farming.

The relationship between soil characteristics and fertilization is often overlooked, and there are interactions between different elements of its composition that heavily affect the efficiency of nutrient absorption by the roots (Jones, 2012). In this respect, the imbalanced N–P–K fertilization in the low N treatment was taken into account before setting the fertilization program by evaluating the possible impaired plant availability of other macronutrients. However, only synergistic or zero interactions have been identified in literature between nitrogen and phosphorus or potassium. Antagonistic effects are mostly found between divalent cations (Rietra et al., 2017). In addition, the soil texture influences the development of the roots and their degree of absorption (De Pascale et al., 2016). A clay-loam soil, as in the present work, would enhance root efficiency in exploring the soil for nutrients as it represents a well-balanced soil with intermediate compaction degree, which avoids rapid loss of nutrients and water and allows proper aeration (Tracy et al., 2013). Our experiment was carried out in an officially recognized area for ‘de penjar’ tomato cultivation. In this work, soil analysis showed slight salinity, which is optimal for ‘de penjar’-type cultivation (Conesa et al., 2020), and high organic matter concentration, which is known to have a positive impact on nutrient availability and reducing soil compaction (Metzger and Yaron, 1987). No nutrient deficiencies were found in the soil of study. Contrarily, concentrations of K, Ca^2+^, and Mg^2+^ were excessive, which could result in salt formation causing antagonism between ions (e.g., Na^+^ vs. K^+^, Cl^–^ vs. NO_3_^–^) and mineral imbalance with negative impact on plant growth (Jones, 2012). In addition, very high P concentrations were found in soil, which will eventually be washed out through the soil, contributing to pollution and eutrophication of waters. These data prove that there is scope for reducing, at a large extent, the supply of these nutrients in fertilization of ‘de penjar’ tomato.

### Variation Observed

Considerable phenotypic variation was observed in our collection for most of the traits evaluated, which is in agreement with the large genetic diversity described for ‘de penjar’ tomato in other works (Casals et al., 2012; Cebolla-Cornejo et al., 2013; Esposito et al., 2020). Genotyping data of our collection would have been relevant to confirm at the genetic level the high diversity we have found. However, since we mostly evaluated quantitative traits with polygenic control, a larger number of accessions would have been needed for a robust “Genome Wide Association Study” (GWAS) (Korte and Farlow, 2013). The specific effect of the *alc* mutation on the traits investigated could be studied by means of crosses between parents carrying the *alc* mutation and its corresponding wild allele. The PCA confirmed the wide variation observed, visually represented with the different accessions studied scattered all over the score plot. This supports the definition of ‘de penjar’ tomato by Conesa et al. (2020) as a “population of landraces,” in which the *alc* mutation is introgressed into different genetic backgrounds (Casals et al., 2012), maintaining high heterogeneity within the ‘de penjar’ type.

The higher value of phenotypic than genotypic variation for every trait analyzed shows an important environmental effect, especially for fruit bioactive and quality compounds. This is in agreement with previous studies that reported tomato quality traits being highly polygenic, strongly influenced by environmental conditions and showing low heritability (Causse et al., 2003). Fruit mean weight showed the highest broad-sense heritability estimate (H^2^), followed by malic acid content and citric:malic acid ratio, which was in line with them showing the highest genotypic variation coefficient (CV_*G*_). In these terms, similar results were reported in other works. Morphology traits in tomato, such as fruit weight and skin color, are known to have higher values of heritability than fruit quality traits or yield (Figàs et al., 2018a). Panthee et al. (2012) also observed that acid traits showed higher heritability than sugar and soluble solid content. Contrarily, lycopene, total carotenoid, and β-carotene contents had the lowest H^2^ and the highest phenotypic variation coefficient (CV_*P*_) in the collection of the present study. Both H^2^ and CV_*P*_ estimates for lycopene and total carotenoid content showed values of 0. We attribute this phenomenon not to the absence of genetic variation in our collection but to a strong interaction G × N, meaning that different trends in the response to increasing or decreasing N inputs were observed among genotypes. Panthee et al. (2012) also found a similar interaction genotype × environment effect resulting in low heritability for lycopene in tomato. Both heritability and a strong interaction G × N constitute relevant information for breeding and selection (Panthee et al., 2012; Figàs et al., 2018b). While high heritability estimates would make more efficient the selection of genotypes expecting the same performance under different N supply conditions, having a strong interaction G × N would allow breeders to select varieties with the best response under certain conditions, in our case, low N.

### Effects of Low Nitrogen Inputs on Traits Evaluated

Yield is one of the most valuable traits for growers, and it is directly correlated with N availability for plants (Zhang et al., 2015). In the present work, no significant differences were found between the two N treatments regarding average yields for any of the varieties evaluated. Our results suggested that, although N supplied in the high N treatment could not be considered excessive in a detrimental way to yield, the N availability in the low N treatment was suitable for an optimal plant growth and for obtaining similar yields. More plant growth parameters would have been needed for better support of our statement. Most of the studies testing tomato cultivars in soil with different rates of N fertilization found that yield increased linearly with N input but reached a plateau where it became insensitive to more N fertilization levels (Elia and Conversa, 2012; Djidonou et al., 2013). However, among those studies, the minimum N fertilization level maximizing tomato yield was at least 168 kg ha^–1^ in open field (Djidonou et al., 2013), while for the ‘de penjar’ collection studied, we found that even with only 49 kg ha^–1^ of N fertilization, no differences were obtained compared to the standard fertilization. In addition, data of SPAD were similar to those of the plant yield. More data about plant morphological changes would be appropriate to robustly evaluate the effect of the LN treatment on plants, like plant biomass, plant height, leaf size, or root morphology (Hermans et al., 2006; Freschet et al., 2018). In particular, it would have been of interest to have plant biomass data to evaluate nitrogen use efficiency parameters. However, SPAD values have been widely used for evaluating plant N status in crop management, as leaf chlorophyll content is closely related to leaf N content, and a reduction in these values have been already reported for tomato under N fertilization stress (Ling et al., 2011; Padilla et al., 2015; Dunn et al., 2018). Thus, we decided to use this parameter to estimate plant N status for being a simple, nondestructive, and relatively quick measure to take. Fruit mean weight was also not influenced by the N rate in the present study. In fact, Elia and Conversa (2012) and Hernández et al. (2020) suggested that the effects of N inputs on tomato yield were due to changes in fruit load more than fruit mean weight. The external color of fruit was evaluated through lightness, chroma, and hue values, but only the collection average for hue was significantly affected by the N treatment, increasing when low N was applied. According to the HCL color space, this would represent a slight change of color toward orange under low N inputs, possibly due to a slightly higher content of β-carotene under these conditions.

Taste and flavor of ‘de penjar’ tomato is one of the attributes most appreciated by the local consumer, and it is associated with its traditional use (Conesa et al., 2020). Therefore, we considered important to evaluate the effects of lowering the dose of N fertilization on those characteristics. Our results showed that high N rate did not affect significantly either the N or the C content in fruit, probably due to a N redistribution between leaves and fruits in response to an exceeded N supply. In fact, Elia and Conversa (2012) found that increasing N inputs of tomato from 200 to 300 kg ha^–1^ increased leaf N, while N storage in fruits decreased. This was explained by a plant tendency to grow vegetatively rather than reproductively when N availability increases over the demand.

Citric, malic, and total acid contents and titratable acidity in fruits showed no significant differences between N treatments. Our results differed to the ones reported in recent studies for tomato, both under hydroponic and soil cultivation (De Pascale et al., 2016; Truffault et al., 2019), in which higher acidity was found related to an increasing N rate. In addition, our results suggest that citric acid is likely the highest contributor to the fruit acidity in ‘de penjar’ collection, as its content in fruits exceeds by 2.7- to 10.0-fold on average that of malic acid. This is in agreement with the results obtained in the collection evaluated by Casals et al. (2015), which showed nearly the same range of citric:malic ratio (2.4 to 9.3).

Fruit contents in fructose, glucose, total sugar, and total sweetness index were the only composition traits evaluated in this study that showed a significant effect of the N treatment. All of them suffered an average reduction of 10–20% in response to low N inputs. Although the ANOVA did not show significant differences for these traits between N treatments for each accession separately, the existence of a significant effect of the N treatment indicates a tendency toward the reduction of sugar content under low N in the ‘de penjar’ tomato. Results about the N effect on carbohydrates and total soluble solids in fruits are the most controversial among other works in tomato crop. Our results are in agreement with some of them (De Pascale et al., 2016). Others reported the opposite effect (Hernández et al., 2020), while others even found no significant effect of N rate on sugar content (Truffault et al., 2019). According to Hermans et al. (2006), N deficiency in plants may cause an accumulation of starch and sugars in leaves, consequently decreasing their content in fruits and regulating photosynthesis by negative feedback. This could explain our results. Level of sweetness is also related to different proportions of main sugars, with fructose being the sweetest, followed by sucrose and finally glucose. Fructose and glucose are also known to be usually in the same proportion (1:1) in tomato pericarp (Beckles, 2012). However, our results showed mean values of fructose:glucose ratio over 1.0 for every accession and it was not affected significantly by N inputs. Furthermore, a highly significant interaction G × N was observed, which gives scope to select accessions with higher fructose:glucose ratio under low N inputs for breeding purposes.

Since the end of the 20th century, flavor was increasingly understood as a complex parameter not only due to main sugars and organic acids but also due to their ratio, texture, and volatile compounds. Although studies including the different elements of flavor are emerging, it is still a difficult parameter to estimate objectively and not amenable for exhaustive assays for being time-consuming and expensive. Therefore, the horticultural industry has been using indexes highly correlated with flavor and consumer acceptability for selecting and breeding (Beckles, 2012). In tomato, Baldwin et al. (1998) reported that total sweetness index or its ratio to titratable acidity were closer than soluble solid content or its ratio to titratable acidity to their acceptability based on sweetness we perceive (*r* ≥ 0.80). In the present study, while total sweetness index significantly decreased with LN treatment, soluble solid content was not affected. Despite the high correlation between sugar and soluble solid content and that the latter is easier and faster to measure, soluble solid content includes other compounds that do not contribute to sweetness. Thus, it could be interesting to quantify individual sugars (Fernández-Ruiz et al., 2004). On the other hand, as acidity level influences the perception of sweetness, parameters related to sweetness–acidity balance are more likely to correlate with taste preferences than sugar and acid contents alone (Baldwin et al., 1998; Beckles, 2012). In this experiment, a significant decrease in average sugars associated with low N inputs was reflected as a slight reduction of their average ratios with organic acids and acidity-related traits. Remarkably, this reduction was not significant; thus, low N inputs may not change a great deal the taste of ‘de penjar’ tomatoes. On the other hand, taking into consideration the traditional conservation and consumption of the ‘de penjar’ tomato, up to a minimum of 2 months after harvest, having lower sugar content under low N treatment could be detrimental to its taste during postharvest life, since a decrease, first sharply and then more gradual, of sugar content in this type of tomato has been reported at 2 to 4 months postharvest, while organic acids decreased to a lesser degree in the same period (Casals et al., 2015). However, further studies are needed to draw an accurate conclusion, using experienced sensory panels and investigating the postharvest performance of the accessions evaluated, as diversity for these traits has been observed among ‘de penjar’ accessions (Casals et al., 2015).

One possible mitigation of the reduction of sugars under low N conditions would be the use of foliar sprays as a nutritional complement. The main advantage of fertilizing by foliar treatments is its efficiency with a minimal contribution to environment pollution. Recently, applications of 1 mg L^–1^ sodium selenate or 500 mg L^–1^ abscisic acid have been reported to improve fructose, glucose, and vitamin C, in tomato fruits (Barickman et al., 2017; Zhu et al., 2018). On the other hand, the best approach might be an optimized fertilization and irrigation management. As reported by Fullana-Pericàs et al. (2019), ‘de penjar’ tomato local varieties withstand low irrigation conditions with minimum yield losses, and even with enhanced sugar content in fruits. In any case, these are possible paths for further studies about the cultivation of ‘de penjar’ tomato using less N fertilization.

Aspartic and glutamic acids are major amino acids in tomato fruits. Tomato accumulate several-fold higher content of glutamic acid in their fruits than other vegetables such as pepper, onion, or carrot (Haytowitz et al., 2011). On the one hand, aspartic and glutamic acids function as other amino acid precursors in plants, e.g., glutamic acid constitutes the first element of the GABA (γ aminobutyric acid) synthesis, a bioactive molecule of recent interest for its health-promoting potential (Gramazio et al., 2020). On the other hand, those are the only amino acids that are related to taste, especially glutamic acid. In their ionized forms and in the presence of sodium salt, they give the fifth basic taste, umami, known as savory and taste-enhancing (Lioe et al., 2010). Due to its molecular N basis, the amount of amino acid in the plant will depend on the N content of the plant. In the case of aspartic and glutamic acids, no significant differences were found in our ‘de penjar’ collection by reducing nitrogen fertilization to one third of the usual supply.

Regarding bioactive compounds, tomatoes owe their antioxidant power mainly to their vitamin C and carotenoid content (Adalid et al., 2010). Although the effects of lowering N fertilization on these compounds are still not clear (Truffault et al., 2019; Hernández et al., 2020), in our experiment they were not significantly affected by N dosage. Interestingly, vitamin C showed a significant positive environmental correlation to sweetness (soluble solids content, fructose, total sweetness index). A positive correlation between fruit content in sugars and vitamin C has been already reported in other works (Causse et al., 2003) and could be explained by the role of sugars as precursors for vitamin C biosynthesis. On the other hand, sugars also function as signaling molecules in source–sink regulation and as regulators of gene expression, which could also be involved in this correlation (Eveland and Jackson, 2012). These positive correlations could help breeders to identify trends in several compounds measuring just a few easier ones.

### Selection Based on Ideotype and Comparison of Local vs. Commercial Materials

Considering all the traits analyzed in the present work, there are prospects for selecting the best ‘de penjar’ varieties based on an ideotype under low N inputs with the aim to include them in breeding programs or directly cultivate them under those conditions. The desirable attributes pursued in our study would be high yield in the first place; high nitrogen content, due to its direct relationship with protein content; great sweetness, given by high content in sugars (mainly fructose), but moderate content in organic acids resulting in high sweetness:acidity ratio; high glutamic and aspartic acid contents, which would potentiate taste; and great content in antioxidant compounds (vitamin C, carotenoids) due to their reported bioactive role in the human body. In this regard, among the best ten yielding varieties under low N treatment, both MT1 and MO1 seemed to present the best ideotype. In addition, they showed opposite average fruit weight, which could be an advantage in breeding for different markets and maintaining the morphological variability within the ‘de penjar’ type. On the other hand, MO2 showed very good resilience in yield ranking third, besides being the fourth best-yielding variety under low N treatment. Among the 19 out of 44 accessions that showed significant differences between the two N treatments for some of the traits evaluated (lightness of color, fructose:glucose ratio, contents in lycopene, β-carotene, total carotenoids, and carbon), the already mentioned MT1 was very interesting for showing increased average contents of lycopene, β-carotene, and total carotenoids by 43–52% under low N treatment. In addition, TO1 stood out for showing increased mean values under the low N treatment compared to the high N for all the mentioned traits, except for lightness of color. Although TO1 did not rank in the top 10 positions for yield in any of the N treatments, nor for yield resilience, the cultivation and conservation of this local variety could be interesting for future breeding programs aimed at improving fruit quality under low N inputs.

Commercial tomato varieties are the result of the last 50 years of breeding toward high yield, pest resistance, and fruit uniform appearance (Casañas et al., 2017). However, in the last decade, with consumers demanding more tasteful and healthful fruits and agriculture facing the challenge of producing more with fewer resources and with less impact on the environment, local tomato varieties are being “rediscovered” as an important source of variability. Several studies have been carried out in order to properly characterize the ‘de penjar’ tomato (Casals et al., 2012, 2015; Cebolla-Cornejo et al., 2013; Figàs et al., 2015). However, there is a lack of comparison of local vs. commercial varieties for this varietal type. Herein, we have found significant differences for three (soluble solid content:titratable acidity, total sweetness index:titratable acidity, and vitamin C) out of the 29 traits analyzed. For all three traits and both N treatments, local varieties showed a higher average value, which reinforces their appreciation for their organoleptic and nutritional quality.

## Conclusion

The present work provided the first comprehensive characterization of the variability of ‘de penjar’ tomato varieties under contrasting levels of N fertilization. A wide diversity in our collection for agronomical, morphological, and fruit organoleptic and nutritional quality traits was revealed. Our data support the evidence of a current over-fertilization in ‘de penjar’ tomato cultivation. Under the experimental conditions tested, reducing to one third the usual nitrogen supply did not show any significant effect on yield and most of the traits evaluated related to fruit nutritional and organoleptic quality, except for a decrease in soluble sugars. Several varieties showed excellent results under low N supply conditions, being within the best ten yielding varieties with good fruit quality parameters. In addition, the present work highlights the value of local varieties for selection and breeding of ‘de penjar’ tomatoes and enhances their potential as a very useful gene pool for future tomato breeding programs for resilience under restrictive environmental conditions. Further studies on association of genetic and phenotypic data and on postharvest performance under low N fertilization conditions, as well as developing segregating generations for the *alc* mutation, will provide relevant information for the enhancement of the ‘de penjar’ tomato for selection and breeding for resilience.

## Data Availability Statement

The raw data supporting the conclusions of this article will be made available by the authors upon request, without undue reservation.

## Author Contributions

JP, SS, and MP planned the study and supervised the research. ER-M, LA, and RB performed the morphological and chemical composition characterization. AA and ER-M performed the chemical analyses by HPLC. LP-D, CC, ES, MF, RB, LA, and ER-M performed the agronomic characterization. MG-M performed the soil analysis. LP-D, CC, ES, MF, and SS supervised the crops. ER-M and RB curated the data. LP-D and ER-M performed the statistical analyses. ER-M, JP, SS, and MP drafted the manuscript. All authors contributed to the article and approved the submitted version.

## Conflict of Interest

MP was employed by company Meridiem Seeds S.L. The remaining authors declare that the research was conducted in the absence of any commercial or financial relationships that could be construed as a potential conflict of interest.

## Supplementary Material

Supplementary Figure 1 Daily maximum (purple line), average (red line) and minimum (green line) temperatures **(A)**, and radiation (orange area) and pluviometry (blue columns) **(B)** since the transplant, on May 8th 2019, until the end of cultivation, on September 30th 2019.

Supplementary Table 1 Accession name, code used in the present work and geographical origin within Spain or breeding company or institution of the ‘de penjar’ tomato local and commercial varieties used for their evaluation under low and high nitrogen fertilization treatments.

Supplementary Table 2 Mean values and standard error of the soil chemical composition for each soil of both low and high nitrogen fertilization field plots (*n* = 5 × 2 = 10).

Supplementary Table 3 Mean values of the three replicates per accession and treatment (*n* = 3 × 2 = 6) for all the traits evaluated. Acronyms LN and HN refer to low nitrogen and high nitrogen treatment, respectively.
