# Supplementary material for: Variation for Composition and Quality in a Collection of the Resilient Mediterranean ‘de penjar’ Long Shelf-Life Tomato Under High and Low N Fertilization Levels
Source: Front Plant Sci. 2021 Apr 7;12:633957. doi: 10.3389/fpls.2021.633957 (PMC8058473; doi:10.3389/fpls.2021.633957)
Supplement: Supplementary Figure 1 — Daily maximum (purple line), average (red line) and minimum (green line) temperatures (A), and radiation (orange area) and pluviometry (blue columns) (B) since the transplant, on May 8th 2019, until the end of cultivation, on September 30th 2019. [file Image_1.pdf]

Supplementary Material

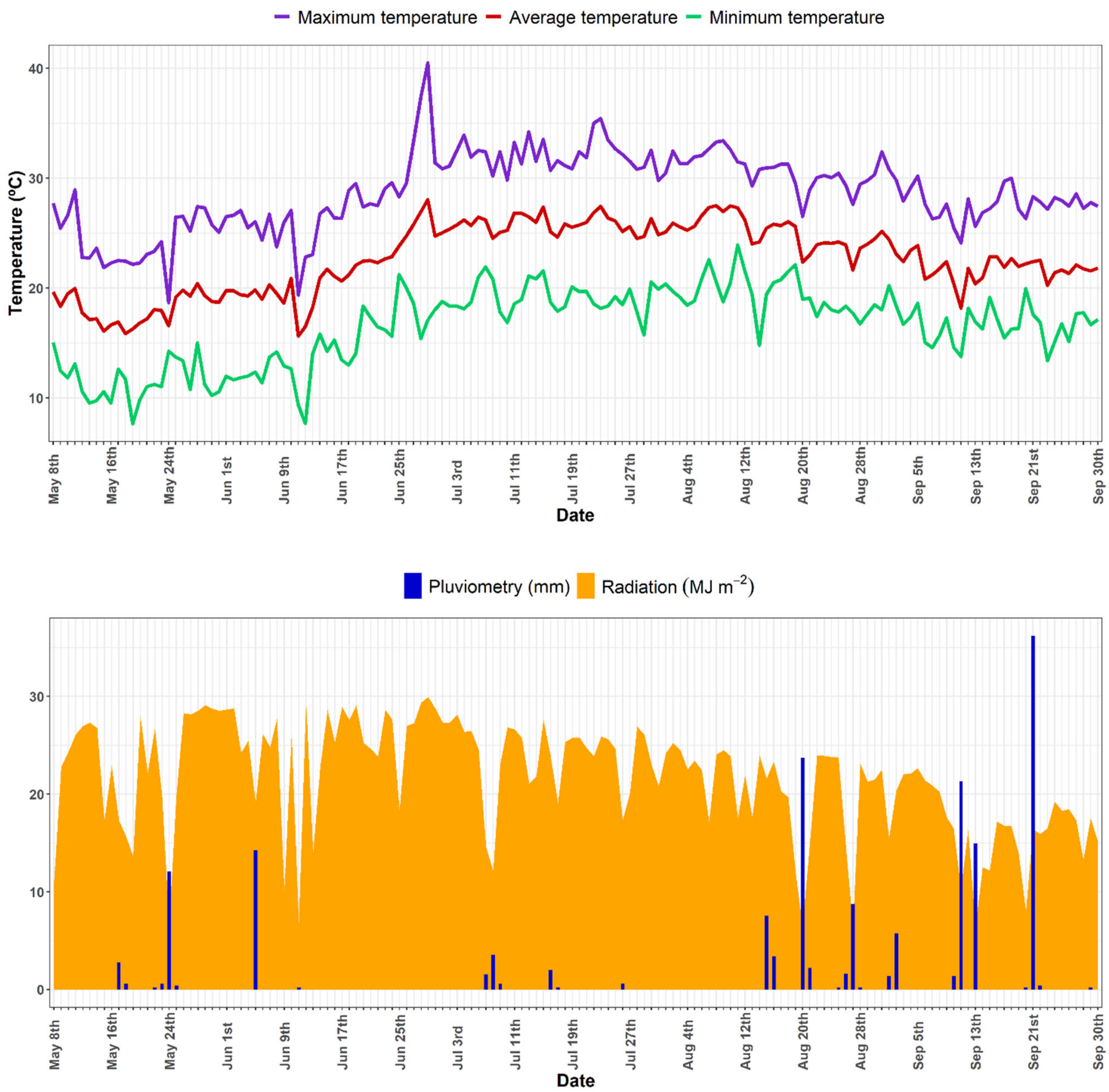

Figure S1. Daily maximum (purple line), average (red line) and minimum (green line) temperatures (A), and radiation (orange area) and pluviometry (blue columns) (B) since the transplant, on May 8th 2019, until the end of cultivation, on September 30th 2019.
