# Supplementary material for: Variation for Composition and Quality in a Collection of the Resilient Mediterranean ‘de penjar’ Long Shelf-Life Tomato Under High and Low N Fertilization Levels
Source: Front Plant Sci. 2021 Apr 7;12:633957. doi: 10.3389/fpls.2021.633957 (PMC8058473; doi:10.3389/fpls.2021.633957)
Supplement: Supplementary Table 1 — Accession name, code used in the present work and geographical origin within Spain or breeding company or institution of the ‘de penjar’ tomato local and commercial varieties used for their evaluation under low and high nitrogen fertilization treatments. [file Table_1.docx]

Supplementary Material

Table S1. Accession name, code used in the present work and geographical origin within Spain or breeding company or institution of the ‘de penjar’ tomato local and commercial varieties used for their evaluation under low and high nitrogen fertilization treatments.

| Accession name | Code | Municipality and province / Breeding company or institution |
| --- | --- | --- |
| *Local varieties* | | |
| SL-ALCALADEXIVERT-1 | AX1 | Alcalà de Xivert, Castelló |
| SL-ALCALADEXIVERT-2 | AX2 | Alcalà de Xivert, Castelló |
| SL-ALCALADEXIVERT-3 | AX3 | Alcalà de Xivert, Castelló |
| SL-ALCORA-1 | AC1 | Alcora, Castelló |
| SL-ALGINET-1 | AG1 | Alginet, València |
| SL-ARANYUEL-1 | AY1 | Aranyuel, Castelló |
| SL-BENLLOCH-1 | BL1 | Benlloc, Castelló |
| SL-CASTELLFORT-3 | CF3 | Castellfort, Castelló |
| SL-CHELVA-1 | CH1 | Chelva, València |
| SL-CINCTORRES-2 | CI2 | Cinctorres, Castelló |
| SL-COCENTAINA-1 | CO1 | Cocentaina, Alacant |
| SL-FANZARA-2 | FA2 | Fanzara, Castelló |
| SL-FIGUEROLES-1 | FI1 | Figueroles, Castelló |
| SL-LAJANA-1 | LA1 | La Jana, Castelló |
| SL-LAJANA-2 | LA2 | La Jana, Castelló |
| SL-LLIRIA-1 | LL1 | Lliria, València |
| SL-LLIRIA-2 | LL2 | Lliria, València |
| SL-MONTAN-1 | MO1 | Montan, Castelló |
| SL-MONTAN-2 | MO2 | Montan, Castelló |
| SL-MONTROI-1 | MT1 | Montroi, València |
| SL-PEGO-1 | PG1 | Pego, Alacant |
| SL-SALZADELLA-2 | SA2 | La Salzadella, Castelló |
| SL-SANTMATEU-2 | SN2 | Sant Mateu, Castelló |
| SL-SONEJA-1 | SO1 | Soneja, Castelló |
| SL-TEULADA-1 | TE1 | Teulada, Alacant |
| SL-TEULADA-2 | TE2 | Teulada, Alacant |
| SL-TORREBAIXA-1 | TO1 | Torrebaixa, València |
| SL-TORREDENDOMENECH-1 | TR1 | La Torre d'en Doménec, Castelló |
| SL-TRAIGUERA-1 | TA1 | Traiguera, Castelló |
| SL-VALLDALBA-1 | VA1 | Vall d'Alba, Castelló |
| SL-VILLAHERMOSADELRIO-1 | VH1 | Villahermosa del Río, Castelló |
| SL-VILLAHERMOSADELRIO-2 | VH2 | Villahermosa del Río, Castelló |
| SL-VINAROS-1 | VI1 | Vinaròs, Castelló |
| SL-VINAROS-2 | VI2 | Vinaròs, Castelló |
| SL-VISTABELLA-1 | VT1 | Vistabella del Maestrat, Castelló |
| SL-VIVER-1 | UV1 | Viver, Castelló |
| SL-XAVIA-1 | XA1 | Xàbia, Alacant |
| SL-XAVIA-2 | XA2 | Xàbia, Alacant |
| SL-XERICA-1 | XE1 | Jérica, Castelló |
| *Commercial varieties* | | |
| MANACOR F1 | C1 | Semillas Fitó |
| PALAMOS F1 | C2 | Semillas Fitó |
| DOMINGO | C3 | Semillas Batlle |
| MALLORQUÍN | C4 | Semillas Batlle |
| JORBA | C5 | Universitat Politècnica de València |
