# Supplementary material for: Variation for Composition and Quality in a Collection of the Resilient Mediterranean ‘de penjar’ Long Shelf-Life Tomato Under High and Low N Fertilization Levels
Source: Front Plant Sci. 2021 Apr 7;12:633957. doi: 10.3389/fpls.2021.633957 (PMC8058473; doi:10.3389/fpls.2021.633957)
Supplement: Supplementary Table 2 — Mean values and standard error of the soil chemical composition for each soil of both low and high nitrogen fertilization field plots (n = 5 × 2 = 10). [file Table_2.docx]

Supplementary Material

Table S2. Mean values and standard error of the soil chemical composition for each soil of both low and high nitrogen fertilization field plots (*n* = 5 × 2 = 10).

| Soil characteristics | Low nitrogen (*n* = 5) | High nitrogen (*n* = 5) |
| --- | --- | --- |
| pH in water | 8.22 ± 0.04 | 8.23 ± 0.12 |
| pH in KCl | 7.38 ± 0.01 | 7.44 ± 0.03 |
| Electrical conductivity (µS cm^-1^) | 302.0 ± 27.5 | 361.0 ± 40.6 |
| Total nitrogen content (g kg^-1^ dm) | 1.67 ± 0.06 | 1.63 ± 0.14 |
| Content in carbonates (g kg^-1^ dm) | 135.4 ± 14.0 | 136.0 ± 18.7 |
| Organic matter content (g kg^-1^ dm) | 36.26 ± 1.45 | 33.43 ± 3.18 |
| Carbon:nitrogen ratio | 12.60 ± 0.25 | 11.90 ± 0.23 |
| P (mg kg^-1^ dm) | 127.2 ± 10.9 | 126.7 ± 13.2 |
| K (mg kg^-1^ dm) | 986.0 ± 56.4 | 947.0 ± 54.2 |
| Ca^2+^ (cmol kg^-1^ dm) | 126.0 ± 10.4 | 125.0 ± 13.5 |
| Mg^2+^ (cmol kg^-1^ dm) | 6.94 ± 0.19 | 6.71 ± 0.28 |
| Fe (mg kg^-1^ dm) | 8.74 ± 0.36 | 7.51 ± 0.57 |
| Zn (mg kg^-1^ dm) | 9.00 ± 0.57 | 7.45 ± 0.88 |
| Cu (mg kg^-1^ dm) | 17.12 ± 1.01 | - 1. ± 2.43 |
